# Effects of Recombinant Spidroin rS1/9 on Brain Neural Progenitors After Photothrombosis-Induced Ischemia

**DOI:** 10.3389/fcell.2020.00823

**Published:** 2020-09-08

**Authors:** Mikhail M. Moisenovich, Denis N. Silachev, Anastasia M. Moysenovich, Anastasia Yu. Arkhipova, Konstantin V. Shaitan, Vladimir G. Bogush, Vladimir G. Debabov, Alexander V. Latanov, Irina B. Pevzner, Ljubava D. Zorova, Valentina A. Babenko, Egor Y. Plotnikov, Dmitry B. Zorov

**Affiliations:** ^1^Department of Biology, Lomonosov Moscow State University, Moscow, Russia; ^2^Laboratory of Mitochondrial Structure and Function, A. N. Belozersky Institute of Physico-Chemical Biology, Lomonosov Moscow State University, Moscow, Russia; ^3^V.I. Kulakov National Medical Research Center of Obstetrics, Gynecology, and Perinatology, Moscow, Russia; ^4^Histology, Embryology and Cytology Department, Peoples’ Friendship University of Russia, Moscow, Russia; ^5^National Research Center “Kurchatov Institute” – GOSNIIGENETIKA, Moscow, Russia; ^6^National Research Center “Kurchatov Institute”, Moscow, Russia; ^7^Institute of Molecular Medicine, I.M. Sechenov First Moscow State Medical University, Moscow, Russia

**Keywords:** scaffold, fibroin, stroke, neural progenitors, mitochondria

## Abstract

The existence of niches of stem cells residence in the ventricular–subventricular zone and the subgranular zone in the adult brain is well-known. These zones are the sites of restoration of brain function after injury. Bioengineered scaffolds introduced in the damaged loci were shown to support neurogenesis to the injury area, thus representing a strategy to treat acute neurodegeneration. In this study, we explored the neuroprotective activity of the recombinant analog of *Nephila clavipes* spidroin 1 rS1/9 after its introduction into the ischemia-damaged brain. We used nestin–green fluorescent protein (GFP) transgenic reporter mouse line, in which neural stem/progenitor cells are easily visualized and quantified by the expression of GFP, to determine the alterations in the dentate gyrus (DG) after focal ischemia in the prefrontal cortex. Changes in the proliferation of neural stem/progenitor cells during the first weeks following photothrombosis-induced brain ischemia and *in vitro* effects of spidroin rS1/9 in rat primary neuronal cultures were the subject of the study. The introduction of microparticles of the recombinant protein rS1/9 into the area of ischemic damage to the prefrontal cortex leads to a higher proliferation rate and increased survival of progenitor cells in the DG of the hippocampus which functions as a niche of brain stem cells located at a distance from the injury zone. rS1/9 also increased the levels of a mitochondrial probe in DG cells, which may report on either an increased number of mitochondria and/or of the mitochondrial membrane potential in progenitor cells. Apparently, the stimulation of progenitor cells was caused by formed biologically active products stemming from rS1/9 biodegradation which can also have an effect upon the growth of primary cortical neurons, their adhesion, neurite growth, and the formation of a neuronal network. The high biological activity of rS1/9 suggests it as an excellent material for therapeutic usage aimed at enhancing brain plasticity by interacting with stem cell niches. Substances formed from rS1/9 can also be used to enhance primary neuroprotection resulting in reduced cell death in the injury area.

## Introduction

Acute focal brain damage can lead to impaired or lost cognitive, sensorimotor, and visceral functions. The main physiological mechanism of functional recovery of the brain after the damage is neuroplasticity that is, the ability of neuronal tissues to undergo adaptive changes, both at the structural and functional levels—from molecular and cellular changes to global rearrangements of the neural network. During reparation of other tissues, posttraumatic central nervous system (CNS) responses can be divided into three consecutive phases: cell death and inflammation, cell proliferation, and tissue remodeling. Some of these events such as cell death, recruitment of inflammatory and immune cells, the formation of a fibrin-collagen mesh, angiogenesis, and fibroblast proliferation, reflect classic responses to damage in any tissue, whereas others such as infiltration into the injury area of microglia cells, migration of neuronal progenitor cells, and the formation of a compact astroglial scar, are specific and unique to the CNS (Burda and Sofroniew, 2014). Currently, in experimental studies of acute cerebral pathologies, therapeutic strategies are based on approaches of primary neuroprotection aimed at reducing cell death in the damaged loci. However, despite the effectiveness of these approaches proven in preclinical studies, their translation into clinical practice is very difficult because of failures at the level of clinical trials (Moretti et al., 2015). The difficulty of implementation of primary neuroprotection is the limited length of the therapeutic window, which cannot go beyond 5–6 h after damage. On the other hand, approaches aimed at enhancing brain plasticity, i.e., those considering effects on the damaged brain independent on the pharmacologic therapeutic window, such as stem cell or extracellular vesicle transplantation (Bang and Kim, 2019; Borlongan, 2019), or scaffold transplantation (Gopalakrishnan et al., 2019) have shown high therapeutic efficiency.

Adult animal brain is known to have stem cell niches in the ventricular–subventricular zone and the subgranular zone in which new neurons and glial cells are continuously generated from neural stem/progenitor cells (Lazutkin et al., 2019; Obernier and Alvarez-Buylla, 2019). In the subgranular zone of the hippocampus, after exiting the cell cycle, new generations of cells locally migrate to the granular cell layer and turn into mature granular neurons (Lazutkin et al., 2019). There is also evidence of the presence of additional niches of neural progenitor cells associated with vessels, in particular with pericytes and such regionally activated stem cells also contribute to neural regeneration (Shimada et al., 2010, 2012; Nakagomi et al., 2011; Nakata et al., 2017). The balance between stem cell proliferation/differentiation in a cell niche depends on both intercellular interactions and the molecular environment (Obernier and Alvarez-Buylla, 2019). Pathological processes in the brain caused by ischemia or traumatic brain injury can affect the mechanisms of neurogenesis in stem niches (Arvidsson et al., 2002; Gao et al., 2009; Benner et al., 2013; Faiz et al., 2015) including the proliferation of neural stem/progenitor cells, which can be a potential resource for the restoration of damaged nerve tissue. However, in the case of natural regeneration, very few newly created neurons maturate and survive, which may be due to the lack of signals that stimulate the growth of neurites and the suboptimal availability or arrangement of subcellular machinery to enable growth cone reformation and axonal elongation (Bradke et al., 2012). In several studies, it has been shown that various biologically active compounds can enhance the proliferation, differentiation, migration, and subsequent survival of newly formed neuronal cells. For example, fibrinogen is a regulator of neural stem/progenitor cells–derived astrogenesis from the subventricular niche via the BMP receptor signaling pathway following injury (Pous et al., 2020).

The process of choosing strategies for restoration of brain function requires an understanding of how exactly neuroplastic processes occur in the brain in response to trauma and subsequent insertion of the scaffold into the brain lesion. The most modern and informative approach is the use of animal lines with a special reporter, in which stem and progenitor cells carry a genetically encoded construct associated with the expression of a specialized protein typical for this cell type. The most common marker used is nestin which is expressed in the brain stem cell niches of embryos and adult mammals, and it is not found in differentiated cells of neural tissue (Enikolopov et al., 2015; Mignone et al., 2016).

It became clear that the adult brain has a greater neuroplastic potential than previously thought (Chen et al., 2014); however, its ability to regenerate is still quite limited and unable to compensate for the loss of cells and to remodel the extracellular matrix in acute injuries such as focal traumatic injury and ischemic stroke (Moreau et al., 2012). Unlike healing of skin wounds and during other tissue injuries, when the CNS is damaged in the trauma area, there is no anatomical substrate, such as granulation tissue, which can support the migration of progenitor brain cells from stem cell niches to fill cell-free spaces. Thus, to provide the cells with an anatomical substrate for migration and axon guidance, additional scaffolds must be inserted into the injury area.

That is why a promising approach to restoring brain function after an injury can be the introduction of scaffolds supporting intrinsic neurogenesis and/or of those containing various substances that activate the proliferation of resident neural progenitor cells (Nih et al., 2018). Additionally, the matrix can act as a chemoattractant and/or as structural support providing migration of progenitor cells to the area of damage (Volkov et al., 2020). At this time, several compounds have been proposed as scaffolds for the regeneration of nerve tissue which differ in both their composition and three-dimensional structure (Wang et al., 2018). Structural silk proteins from silkworms and spiders are considered as the most promising materials used for neural tissue engineering. They have a unique combination of physical and chemical properties and biological activity that ensure their biocompatibility and high regenerative activity (Magaz et al., 2018; Johari et al., 2020). Substrates based on silk fibroin from *Bombyx mori* are able to support adhesion, viability, and neuritogenesis of neuronal cells in different model systems (Hopkins et al., 2013). Some proteins of the spidroin family could be one of the promising agents for brain recovery after a stroke because of the expression of multiple repeats of a GRGGL sequence recognized by neural progenitors (An et al., 2015). Films made of recombinant spidroins have a more suitable surface charge and substrate stiffness for supporting the growth of primary rat cortical neurons than coatings made of fibroin and polylysine (An et al., 2015). In another study, Lewicka et al. (2012) showed that films made of 4RepCt support proliferation and neuronal differentiation of neural stem cells. Also, earlier it has been shown that the ability of a combined matrix containing recombinant analogs of spider dragline silk proteins–spidroin 1 (rS1/9) and spidroin 2 (rS2/12), polycaprolactone, and platelet-rich plasma supports the growth and neuronal differentiation of reprogrammed human nerve progenitor cells (Baklaushev et al., 2019).

Previously, we have explored the biological properties of substrates generated from the recombinant protein rS1/9 which is an analog of spidroin 1 from *Nephila clavipes*. The rS1/9 molecule has a molecular mass of 94 kDa and consists of nine repeating monomers, each of which contains four sequences called primary repeats (so-called initial repeats). Each of these repeats is enriched with GGL, GGY, and GGQ triplets and contains 1 polyalanine cluster consisting of five to eight amino acid residues. These repetitions are identical to the primary repeats residing in natural spidroin 1. Polyalanine clusters form β-sheets, which, in turn, form crystallites that provide a unique stability to the materials based on spidroins (Bogush et al., 2009). This protein also contains 18 repeats of the NCAM-binding sequence GRGGL, which is a signal for binding neuronal cells. Spidroin rS1/9 has been shown to promote cell proliferation, with both vascular and nerve sprouting inside three-dimensional spidroin structures (Moisenovich et al., 2012).

In this study, we used a transgenic reporter nestin–green fluorescent protein (GFP) expressed in a mouse line, in which neural stem/progenitor cells are easily visualized and quantified to assess the alterations in the dentate gyrus (DG) after focal ischemia in the prefrontal cortex. The recombinant analog of *N. clavipes* spidroin 1 rS1/9 with 18 GRGGL repeats was used to prepare microgels which was introduced into the damaged area of the mouse brain. We examined changes in the proliferation of neural stem/progenitor cells during the first weeks following photothrombosis-induced brain ischemia and also *in vitro* effects of spidroin rS1/9 in rat primary neuronal cultures.

## Materials and Methods

### Scaffolds and Microparticles Assembly

#### Formation of Films

Using the protocols described earlier regenerated fibroin was obtained and later lyophilized (Nosenko et al., 2018). Recombinant protein rS1/9 produced by yeast cells carrying gene rS1/9 was isolated from lysed cells, purified by ion-exchange chromatography in fast protein liquid chromatography up to 95% purity and lyophilized. Fibroin or rS1/9 was dissolved in hexafluoroisopropanol (up to a concentration of 20 mg/mL). One hundred microliters of this solution was pipetted evenly onto a 24-mm cover glass and left for 2 h at room temperature to vaporize hexafluoroisopropanol. The resulting films were incubated for 24 h in 96% ethanol. The films were stored in 96% ethanol in a sealed container at +4°C, and before use, they were washed five times in phosphate-buffered saline (PBS).

#### Generation of Microparticles

Microparticles based on recombinant spidroin rS1/9 with sizes of 100 and 300 μm were obtained as described in Nosenko et al. (2018). Microparticles were stored in 70% ethanol and washed five times in PBS prior to use.

#### Scaffolds Surface Morphology Analysis

Preparation of films and microparticles for scanning electron microscopy (SEM) was performed according to standard protocol. Briefly, films or microparticles were fixed overnight using 2.5% glutaraldehyde in 0.1 M cacodylate buffer at +4°C. The samples were then washed three times in 0.1 M cacodylate buffer at pH7.2 for 5 min, followed by dehydration in a series of ethanol solutions with increasing concentrations and acetone (Chemmed, Russia). After critical point drying using Hitachi critical point dryer HCP-2 (Hitachi, Ltd., Japan), films or microparticles were metallized with a 20-nm-thick platinum layer using Ion Coater IB3 (Eiko Engineering Co., Japan). The resulting samples were analyzed with Camscan S2 microscope (Cambridge Instruments, United Kingdom) at 10-nm resolution and operating voltage −20 kV. Images were obtained using MicroCapture software (SMA, Russia).

#### Preparation of Conditioned Culture Medium by rS1/9

The sterilized rS1/9 microparticles were placed into 24-well plates, and 1 mL of cell culture medium was added to each well. The culture growth medium contained Neurobasal medium A (Gibco, United States) containing 2% serum-free supplement NeuroMax (PanEco, Russia) and 0.5 mM L-glutamine (PanEco). Microparticles were incubated for 1 week at 37°C and 5% CO_2_. Then, medium with microparticles was transferred to sterile microcentrifuge tubes and centrifuged at 16,000 *g* for 30 min. The supernatant was collected and used immediately in the tests.

#### rS1/9 Hydrolysis

The 30 mg/mL rS1/9 microparticles were exposed to trypsin (PanEco) or thrombin (Instrumentation Laboratory Company, Bedford, MA, United States) at 37°C for 2 days, and proteolytic activities were inactivated by supplemented fetal bovine serum (FBS). The solution with microparticles was transferred to sterile microcentrifuge tubes and centrifuged at 16,000 *g* for 30 min. The supernatant was collected and used immediately in the tests.

### Cell Culture Experiments

All experimental procedures conformed to the Guidelines for Proper Control of Animal Experiments approved by the local ethics regulations. Primary cultures of neurons from the hippocampus or cortex were obtained from brains of Wistar rats (hippocampal cells) or BALB/c murine (cortical cells) embryos (E18). The cell suspension was obtained according to the previously published method (Brewer, 1995). Cortical cells were plated on fibroin (FB) or rS1/9 films with a density of 10,000 per 1 cm^2^. The cells were allowed to sediment for 1 h at 37°C and 5% CO_2_, and then non-attached cells were removed, and 1.5 mL of culture medium (Neurobasal medium A containing 2% serum-free supplement NeuroMax and 0.5 mM L-glutamine) was added. Hippocampal cells were plated on glass coated with poly-L-lysine (0.5 mg/mL). The cells were allowed to sediment for 1 h at 37°C and 5% CO_2_, and then non-attached cells were removed, and 1.5 mL of Neurobasal medium A containing 2% serum-free supplement NeuroMax and 0.5 mM L-glutamine or conditioned with rS1/9 Neurobasal medium A containing 2% serum-free supplement NeuroMax and 0.5 mM L-glutamine were added. For cocultivation of microparticles and hippocampal cells, suspension of microparticles was placed in the Transwell cell culture plate inserts (Corning, United States) with pore size 0.4 μm. For experiments, cells were used at day 7 (cortical cells) or days 1, 4, and 8 (hippocampal cells) in culture.

For neural progenitor cell isolation, hippocampal tissues were cut into 1-mm^3^ tissue blocks. Trypsin/EDTA (0.25%; PanEco) was used to digest the tissue at 37°C for 7 min. The digested tissue solution was collected and placed in a 15-mL centrifuge tube, and Dulbecco modified Eagle medium/F12 (DMEM/F12; 1:1; PanEco) containing 1% hiFBS (HyClone) was added to stop the digestion. Centrifugation was performed at 300 *g* for 4 min. The supernatant was discarded, and the cell suspension was resuspended using DMEM/F12 culture media supplemented with 2% NeuroMax (PanEco), 10 ng/mL basic fibroblast growth factor (PanEco), and 0.5 mmol/L Glutamax (Gibco; Thermo Fisher Scientific, Inc., United States). After 12 h, the medium was changed. After reaching 70–80% confluency, the cells were passaged using trypsin/EDTA (0.05%; PanEco). The second passage was seeded on cover slides coated with poly-L-lysine and cultured with the presence of thrombin- or trypsin-digested rS1/9 for 3 days. For BrdU incorporation assay cells were treated with BrdU at final concentration 10 μM for 4 h before fixation.

### Immunofluorescence of Neural Cells

Immunofluorescence was used to visualize cortical cells grown on FB and rS1/9 films for 7 days, as well as to analyze the impact of rS1/9 conditioned medium or presence rS1/9 microparticles on hippocampal cells growth. In both cases, samples were fixed with 4% paraformaldehyde and treated with 0.1% Triton X-100 in PBS for 10 min. Then samples were incubated with 0.1% Tween 20, 0.3 M glycine, and 1% bovine serum albumin (BSA) in PBS for 2 h.

The cortical cells were labeled with anti-neural cell adhesion molecule (NCAM) monoclonal antibodies (56C04; Thermo Fisher Scientific) at 1:200 dilution in 0.1% Tween 20, 0.3 M glycine and 1% BSA in PBS and incubated overnight at 4°C and then labeled with rabbit anti–mouse immunoglobulin G (IgG) (H + L) secondary antibodies conjugated with Alexa Fluor^®^ 546 (1:1,000; Thermo Fisher Scientific). To detect β3-tubulin, anti–β3-tubulin antibody conjugated with Alexa Fluor^®^ 488, was used (1:800; BioLegend). For CD31 detection, sections were incubated with Alexa Fluor 667 conjugated anti-mouse CD31 antibodies (1:300, clone MEC113; BioLegend). Cell nuclei were visualized with Hoechst 33342 (Thermo Fisher Scientific). The images were obtained using an Eclipse Ti-E microscope with an A1 (Nikon Corporation, Japan) confocal module and a CFI Plan Apo VC 20×/0.75 objective. Image analysis was performed using NIS-Elements and ImageJ software.

The hippocampal cells were labeled with antinestin antibody (Rat-401; BioLegend) at 1:200 dilution in 0.1% Tween 20, 0.3 M glycine and 1% BSA in PBS and incubated overnight at 4°C and then labeled with rabbit anti–mouse IgG (H + L) secondary antibody conjugated with Alexa Fluor^®^ 546 (1:1,000; Thermo Fisher Scientific). Cell nuclei were visualized with Hoechst 33342. Images were captured on an Eclipse Ti-E microscope with the A1 confocal module (Nikon Corporation) and a Plan fluor 40×1.3 objective. Image analysis was performed using NIS-Elements and ImageJ software.

The neural progenitor cells were incubated overnight with anti-BrdU antibodies (1:250; Abcam, United States). Unbound antibodies were washed five times with a mixture of PBS/1% FBS/0.1% Tween-20. Samples were treated for 1 h at room temperature with Cy3 conjugated secondary polyclonal antibodies (goat anti–rat IgG H + L, 1:750). Nestin was visualized using primary antinestin antibodies (Rat-401; BioLegend) at 1:200 dilution in 0.1% Tween 20, 0.3 M glycine, and 1% BSA in PBS and incubated overnight at 4°C followed by labeling with rabbit anti–mouse IgG (H + L) secondary antibodies conjugated with Alexa Fluor^®^ 488 (1:1,000; Thermo Fisher Scientific). Nuclei were counterstained with Hoechst 33342. Images were captured on an Eclipse Ti-E microscope with the A1 confocal module (Nikon Corporation) and CFI Plan Apo VC 20×/0.75 objective.

### Nestin-Based Reporter Transgenic Mice

C57BL/6 mice were bred at our facility and used with a 12-h light cycle at a constant temperature (22°C ± 2°C) with *ad libitum* access to food and water. The nestin-GFP mouse line was generated as described (Mignone et al., 2016) and kindly provided by Grigori Enikolopov. The experiments were conducted in accordance with the ethical standards and recommendations for accommodation and care of laboratory animals covered by the Council Directives of the European community 2010/63/EU on the use of animals for experimental studies. The animal protocols were approved by the institutional animal ethics committee (protocol 5/18 from May 14, 2018).

### Induction of Photothrombotic Infarcts

Young male mice (17 ± 3 g) were used for the experiments. Focal cortical infarct was induced using the photothrombosis model as described by Watson et al. (1985) with modification. Under isoflurane anesthesia (2–2.5% in air) by SomnoSuite^®^ system (Kent Scientific Corporation, Torrington, CT, United States), a mouse was placed in a stereotactic frame, and the skull was exposed through a midline incision cleared of connective tissue and dried. The rose Bengal dye (3% solution, 40 mg/kg; Sigma–Aldrich) dissolved in saline was injected into the jugular vein. A green light source (SDLaser 301) producing a 3-mm-diameter light beam was positioned on the region corresponding to the right prefrontal cortex. After 5 min of the dye injections, the brain was illuminated through the intact skull for 10 min. Rectal temperature was kept constant at 37.0°C ± 0.2°C using a heating pad. The animals were divided into following groups: control (*n* = 4), intact mice; Isch + saline (*n* = 12), mice with stroke induction that received intracranial (i/c) injection of saline; Isch + rS1/9 (*n* = 12), mice with stroke induction that received i/c injection of rS1/9 microparticles.

One day after photothrombotic stroke induction, the mice were placed in a stereotaxic frame, and under aseptic conditions, the sutures were removed, and the skull was exposed. In the ischemia + rS1/9 group of animals, suspension of spidroin microparticles in saline was transplanted into a single point in the center of lesion area of the prefrontal cortex using a Hamilton syringe with a needle (Microliter 702 LT). The stereotaxic coordinates for the injection of microparticles into the prefrontal cortex were as follows: +1.6 mm anterior to bregma, and 2.1 mm ventral to the skull surface. The syringe needle was inserted up to a depth of 1.0 ± 0.2 mm through the hole in the skull. The solution was then infused in a total volume of 2 μL at a rate of 0.25 μL/min using a microscrew in order to reduce the pressure on the surrounding tissue. When the solution was infused, the needle was pulled out with a rate of 0.25 mm/min. Group of mice “Isch + saline” received the same volume of saline.

### Dissection of Hippocampal DG

Dissection of the hippocampal DG was made as described by Hagihara et al. (2009).

### Magnetic Resonance Imaging Studies of the Brain Damage

Infarct volume was quantified by analyzing brain magnetic resonance imaging (MRI) scans obtained 7 days after the stroke induction as described previously (Silachev et al., 2009) on a 7-T magnet (Bruker BioSpec 70/30 USR; Bruker BioSpin, Ettlingen, Germany) using 86-mm volume resonator for radiofrequency transmission and a phased array mice head surface coil for a reception. Before scanning, the animals were anesthetized with isoflurane 2–2.5% in a mixture of oxygen and air. Mice were placed in a prone position on a water-heated bed. The heads of the mice were immobilized using a nose mask and masking tape. The imaging protocol included a T2-weighted image sequence (time to repetition = 4,500 ms, time to echo = 12 ms, slice thickness = 0.5 mm).

### Flow Cytometry Assessment of Mitochondrial Transmembrane Potential

Comparative analysis of mitochondrial transmembrane potential in the cells was undertaken using a CyFlow cytometric analyzer (FACS Canto II; Becton Dickinson, BD, United States). The hippocampal DG was isolated as described above and placed in DMEM medium at room temperature in a Petri dish. The DG was dissected, and the tissue was incubated for 15 min in trypsin/EDTA (0.05%/0.02% wt/vol in PBS at 37°C). The cells were rinsed twice with PBS and once with DMEM medium without bicarbonate, dissociated by Pasteur pipette in DMEM, pelleted by centrifugation (210 g for 2 min at 21°C) and resuspended in DMEM. A cell suspension containing 1 × 10^5^ cells was divided into two samples: unstained cells used as a negative control and loaded with tetramethylrhodamine ethyl ester (TMRE). The cell suspension was stained with 100 nM TMRE for 60 min at room temperature; 543-nm laser excitation was used, and emission beyond 560 nm was collected for evaluation of TMRE fluorescence. In total, 200,000 cells per sample were taken; each experiment included the usage of three animals. Target cells were allocated on dot plots based on the values of side and forward light scattering, as well as the intensity of TMRE fluorescence. The results were analyzed using FACS Diva 6.1 (Becton Dickinson) and Kaluza 2.1 (Beckman Coulter, United States) software.

### Tissue Processing

Mice were deeply anesthetized with an overdose of chloral hydrate. Brains were excised after 4 and 7 postoperative days, fixed in 4% formaldehyde with PBS and sliced using a VibroSlice microtome (World Precision Instruments) into 100-μm-thick sections. The brain slices were studied using an LSM510 laser scanning confocal microscope (Carl Zeiss, Jena, Germany). Fluorescence analysis was performed in glass-bottom dishes with excitation at 488 and 543 nm and emission collected at 500 to 530 nm and >560 nm, respectively.

### BrdU Administration

BrdU (100 mg/kg body weight) was administered by intraperitoneal (i.p.) injection. BrdU was diluted in sterile PBS to make a solution of 10 mg/mL.

### Assessment of Cell Proliferation by BrdU Incorporation

For BrdU immunodetection, antigen retrieval was performed before blocking: the samples were kept overnight in Tris-buffer (pH 9) at 65°C and then washed with PBS for 15 min and incubated with 2N HCl for 2 h at 37°C, followed by 10-min neutralization in 0.1 M borate buffer (pH 8.5). Then slices were incubated in 0.1% Tween 20, 0.3 M glycine, and 1% BSA in PBS for 6 h. After blocking, samples were incubated overnight with an anti-BrdU antibody (1:250; Abcam). Non-bound antibodies were washed five times in PBS/1% FBS/0.1% Tween-20. Samples were treated for 2 h at room temperature with Cy3-conjugated secondary polyclonal antibodies (goat anti–rat IgG H + L, 1:750; Thermo Fisher Scientific). Nuclei were counterstained with Hoechst 33342. Images were captured on an Eclipse Ti-E microscope with the A1 confocal module (Nikon Corporation) and a Plan Fluor 40×1.3 objective.

### Western Blot Analysis

Western blotting was used for determination of the levels of βIII-tubulin, glial fibrillary acidic protein (GFAP), and NCAM in hippocampus homogenates prepared 4 days after stroke induction. The brain was isolated immediately after decapitation and cooled in PBS. Hippocampus was used for analysis; it was homogenized in 100 μL of PBS containing 1 mM protease inhibitor [phenylmethylsulfonyl fluoride (PMSF)]. Total protein concentration was measured in samples by bicinchoninic acid kit for protein determination (Sigma, United States). Ten micrograms of total protein in hippocampus homogenate samples were loaded on 12.5% polyacrylamide gel and separated under denaturing conditions. After electrophoresis, gels were blotted 30 min at 1 amp onto polyvinylidene fluoride membranes (Amersham Pharmacia Biotech, United Kingdom), which were blocked for 12 h at 4°C in Tris buffer by 5% non-fat milk and subsequently incubated with primary rabbit anti–βIII-tubulin (Abcam, 1:1,000), mouse anti-GFAP (DAKO, United States, 1:20,000), and mouse anti-NCAM (Invitrogen, United States, 1:400) antibodies. Membranes were processed with secondary antibodies conjugated with horseradish peroxidase (Imtek, Russia, 1:10,000). Detection was performed by a ChemiDoc^TM^ MP imaging system (Bio-Rad, United States) with a WesternBright^TM^ Enhanced Chemiluminescence kit (Advansta, United States).

### Hippocampal GFP Level Assessment

Hippocampus was homogenized in PBS/PMSF buffer. Ten micrograms of total protein in hippocampus homogenate samples were loaded on 12.5% polyacrylamide gel and separated under native conditions. After electrophoresis, gels were visualized by a ChemiDoc^TM^ MP imaging system (Bio-Rad) with Alexa 488 filter.

### Statistical Analysis

Statistical analyses were performed using Statistica 7.0 for Windows (StatSoft, Inc., United States). The normality of the parameter distribution was estimated using the Shapiro–Wilk criterion *W*. All data were presented as means ± standard error of means. Data were compared using Student *t*-test when 2 groups were compared and parametric analysis of variance when more than 2 groups were compared. Differences were considered significant at *p* ≤ 0.05. *p* < 0.05 was considered as statistically significant.

## Results

### The Structural Organization of Scaffolds

**FIGURE 1:**
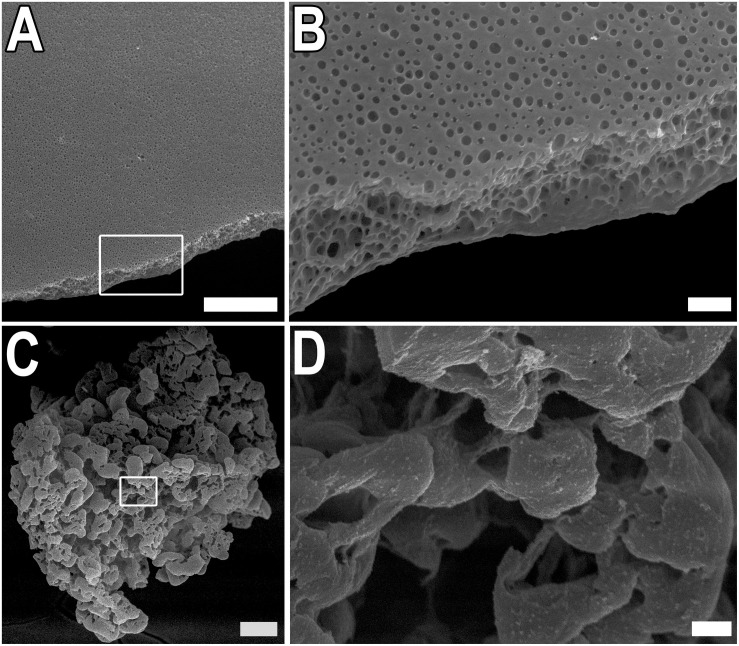
Structure of rS1/9 films and microparticles. **(A,B)** SEM images of rS1/9 films. **(C,D)** SEM images of rS1/9 microparticles. **(A,C)** Scale bar 50 μm. White frames in **(A,C)** indicate the regions, enlarged in **(B,D)**, respectively. Scale bar 3 μm.

Two types of scaffolds were used in experiments – thin films and microparticles based on the recombinant spidroin rS1/9, a recombinant analog of N. clavipes spidroin 1. The surface structure of films and microparticles were analyzed using SEM (Figure 1). The films were prepared by casting rS1/9 (see proper section in Section “Materials and Methods”). The thickness of the films varied from 10 to 20 μm. On the surface and cross-section of the rS1/9 film, spontaneously formed submicrostructures in the form of pores are visible (Figures 1A,B). The pore size varies from 1 to 500 nm on the surface and 10 to 1,000 nm on the cut. Microparticles were obtained as a result of the physical crushing of hydrogel and represent hydrogel particles with a complex surface. Surface elements include nanostructures with a diameter of 100 to 300 nm and microstructures with a size of 10 to 30 μm (Figures 1C,D).

### The Effect of rS1/9 on the Neuronal Cultures Growth

**FIGURE 2:**
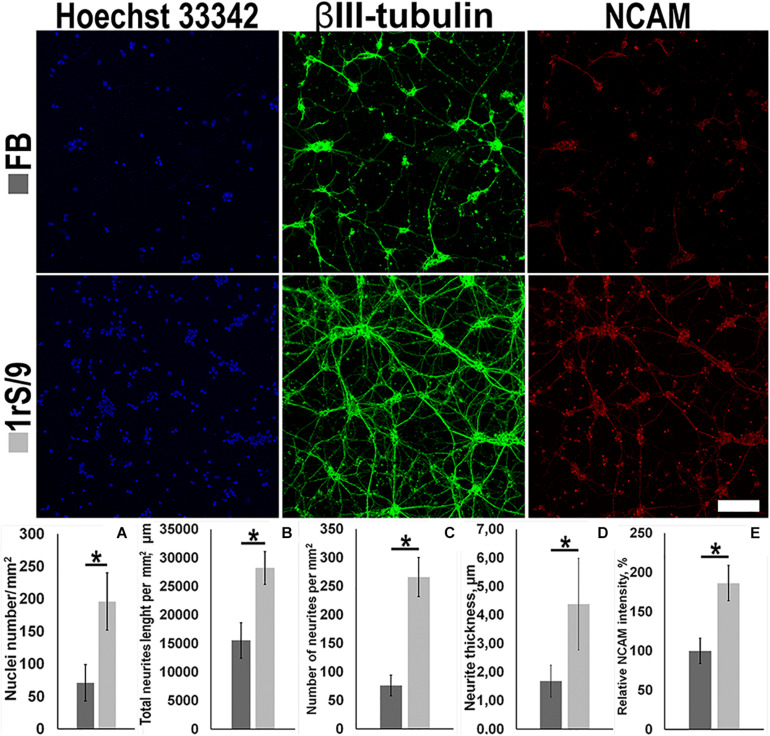
Effect of fibroin films (top) and 1 rS/9 (bottom) on the number of neurons in the mouse cortex and on the morphology of their neurites on the seventh day of cultivation. The cells were treated with antibodies to βIII-tubulin (green) and NCAM (red), and the nuclei were detected by Hoechst 33342 (blue). Scale = 100 μm. **(A)** The number of nuclei per μm^2^, **(B)** total length of neurites per mm^2^, **(C)** number of neurites per mm^2^, **(D)** thickness of neurites, and **(E)** relative intensity values of NCAM. *Significant difference between groups (*p* < 0.05).

To facilitate our studies of the potential effect of rS1/9 material on process formation cortical neurons, we established cultures of embryonic day 18 (E18) murine cortical neurons. The ability of films based on silk proteins to maintain neuronal cell adhesion, as well as the growth of neurites and the formation of a neuronal network, was assessed using immunofluorescence staining with antibodies against β3-tubulin, NCAM, and DAPI. On the seventh day of cultivation of cortical neurons, the number of nuclei on the rS1/9 film was significantly higher compared to FB, 196.5 ± 44.1 and 71.4 ± 28.3, respectively (Figure 2A). β3-Tubulin staining was chosen to evaluate the parameters of neurite outgrowth because it provides bright, specific, and reliable staining of embryonic neurons in the mouse cerebral cortex. To determine the specific parameters of neurite outgrowth affected by rS1/9 films, a comparative morphometric analysis of total neurite length, number of neurites, and the thickness of neurites of cortical neurons cultured on FB and rS1/9 films was performed for 7 days. Compared to cells cultured on a FB-based substrate, total neurite length was significantly greater on rS1/9 (15,536.5 ± 3,102.6 μm and 28,226.6 ± 2,857.7 μm, respectively, Figure 2B). Also, rS1/9 material provided a significant increase in the total number of neurites and their thickness compared to FB was 266.2 ± 34.7 and 76.6 ± 18.3, respectively (Figure 2C) and 4.4 ± 1.6 and 1.7 ± 0.5 μm, respectively (Figure 2D). The relative pixel intensity values of NCAM indicate that NCAM expression is significantly higher on rS1/9 film, compared to FB (186.4% ± 22.6 and 100.0% ± 16.2%, respectively, Figure 2E).

### Effects of rS1/9 on Outgrowth and Branching of Neurites

**FIGURE 3:**
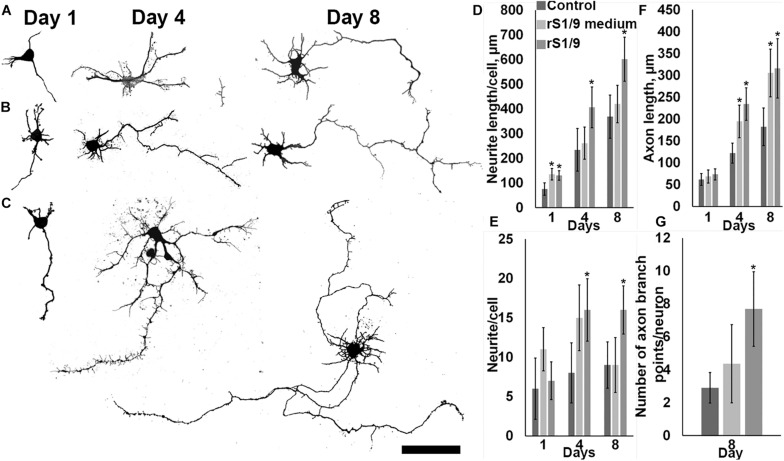
Influence of the presence of rS1/9 microparticles on the cultivation of hippocampal neurons and a conditioned environment on the growth of neuronal sprout. **(A–C)** Binary inverted confocal image of nestin^+^ immunostained cells on days 1, 4, and 8 of cultivation. **(A)** Control, **(B)** hippocampal neurons cultured in a conditioned environment, **(C)** hippocampal neurons cultured in the presence of particles. The scale = 50 μm. **(D)** The length of neurites per cell, **(E)** the number of neurites per cell, **(F)** the length of the axon, and **(G)** the number of axon branch points per neuron. *Significant difference vs. control group (*p* < 0.05).

To determine the effect of rS1/9-based microparticles on the parameters of neurite outgrowth of hippocampal neurons, cultures of E18 rat hippocampal neurons were obtained. Because the injury zone is located at a distance of several millimeters from the hippocampus and direct contact of the injected scaffold with cells of this zone is unlikely, we used a model of *in vitro* cell culturing in the presence of rS1/9 microparticles without direct contact with cells in the Transwell system, as well as in an air-conditioned environment (it contains products of spontaneous hydrolysis of rS1/9 and does not contain rS1/9 itself). To obtain an air-conditioned environment, microparticles rS1/9 were incubated for a week at 37°C in complete growth media under sterile conditions. After this period, the conditioned medium of microparticles was collected and used to quantify neurite outgrowth. To create conditions in which rS1/9 is present in the culture system but does not have direct contact with cells, a suspension of rS1/9 microparticles was placed in a permeable polycarbonate membrane insert. Next, insert containing a suspension of microparticles was transferred to the wells of a 24-well plate with glass coated with poly-L-lysine, after which a suspension of cells was introduced into the well. As a control, cells growing in a standard culture medium on poly-L-lysine–coated glasses were used. The cells were fixed on days 1, 4, and 8 of culture and treated with antibodies to nestin, after which a morphometric analysis of the growth parameters of neurites was performed (Figures 3A–C). On the first and eighth days, the length of neurites of cells cultured in the presence of rS1/9 was significantly higher compared to the control group. On the other hand, the conditioned environment also contributed to the formation of longer neurites on the first day of cultivation, compared to the control, but this difference was not observed on the fourth day. On the first day of cultivation, the total length of neurites was 130.2 ± 19.1 μm (rS1/9), 135.4 ± 23.4 μm (conditioned medium), and 75.9 ± 24.9 μm (control), and on the eighth day of cultivation, 601.4 ± 89.3 μm (rS1/9) and 368.7 ± 87.2 μm (control) (Figure 3D). Cultivation of hippocampal neurons in the presence of microparticles also contributed to an increase in the number of neurites per cell on the fourth and eighth days of cultivation, compared to the control: on the fourth day of cultivation, the number of neurites was 16.19 ± 3.97 (rS1/9) and 8.04 ± 3.81 (control) and on the eighth day – 16.11 ± 3.07 (rS1/9) and 8.57 ± 2.95 (control). There was no statistically significant difference between the group receiving the conditioned environment and the control (Figure 3E). We observed an increase in the length of axons when of hippocampal neurons were cultured in the presence of microparticles starting from the fourth day *in vitro*. At the same time, an increase in the length of the axon in comparison with the control was also observed for cells cultured in a conditioned environment (Figure 3F). Thus, the values of axon length were 234.3 ± 37.1 μm (rS1/9), 194.6 ± 37.3 μm (conditioned medium), and 122.2 ± 22.8 μm (control) on the fourth day of cultivation, and 316.1 ± 67.8 μm (rS1/9), 305.4 ± 54.7 μm (conditioned medium), and 182.2 ± 41.5 μm (control) on the eighth day of cultivation. In addition, the axons of cells cultured in the presence of microparticles had more branching nodes compared to the control group, 7.7 ± 2.3 μm (rS1/9) and 2.9 ± 0.9 μm (control) (Figure 3G).

**FIGURE 4:**
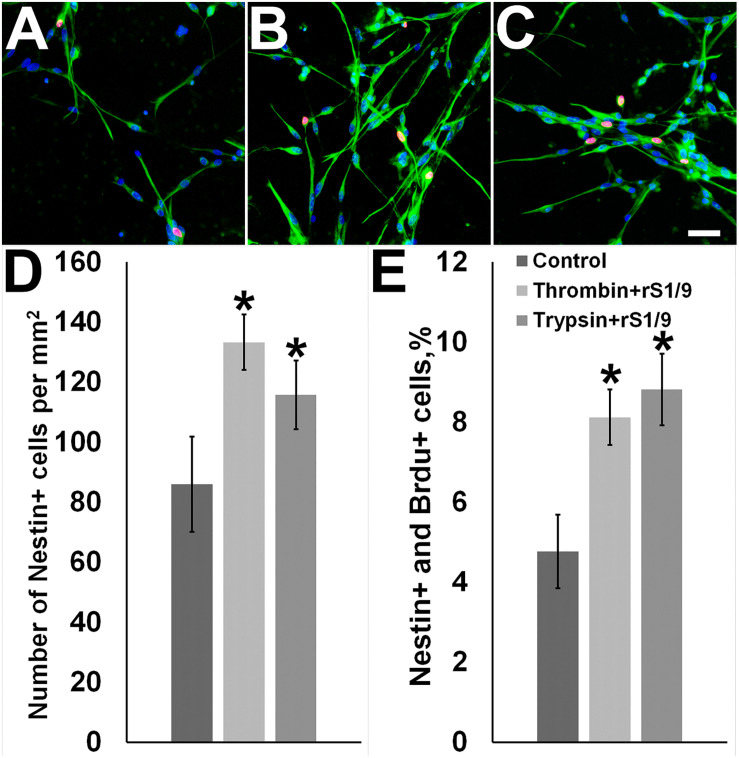
Effects of rS1/9 proteolytic degradation products on cultured neural stem/progenitor cells. **(A)** Control cells, **(B)** cells incubated with thrombin-digested rS 1/9, **(C)** cells incubated with trypsin-digested rS1/9. Neural stem cells were cultured in selective medium and incubated with enzymatically processed rS1/9 for 3 days. The cells were exposed to antibodies to Nestin (green) and BrdU (red), and the nuclei were stained by Hoechst 33342 (blue). Scale = 50 microns. **(D)** The number of Nestin^+^ cells per mm^2^, **(E)** Percentages of Nestin^+^ and BrdU^+^ cells. Values are Mean ± SD of three independent experiments. *Significant difference vs. control group (*p* < 0.05).

To get insight into the mechanisms of action of the rS1/9-conditioned medium (described above), we explored the effect of rS1/9 proteolytic degradation products on the proliferation of neural progenitor cells in culture. It is known that the brain contains several proteases capable to cleave rS1/9 at specific sites. In particular, among these proteases are trypsin IV and thrombin which presence has been demonstrated in the brain tissue (Wang et al., 2008). We exposed rS1/9 microparticles to trypsin or thrombin, added the resulting hydrolysate to the cell culture medium, and kept the culture of neural progenitor cells in the conditioned medium for 3 days. We found that this treatment induced a significant increase in the number of nestin-positive cells in the culture (Figure 4). Certainly, this increase was due to the proliferation of neural stem/progenitor cells, because the majority of nestin-positive cells demonstrated positive BrdU staining.

We treated rS1/9 with trypsin or thrombin and added the obtained hydrolysate to the cell culture media. The addition of the proteolyzed spidroin caused a sharp increase in the number of nestin-positive cells in culture (Figure 4). This increase was associated with the proliferation of neural stem/progenitor cells, because many nestin-positive cells were also positively stained for BrdU.

### Effects of rS1/9 on Neural Progenitors Proliferation in the Hippocampal DG

**FIGURE 5:**
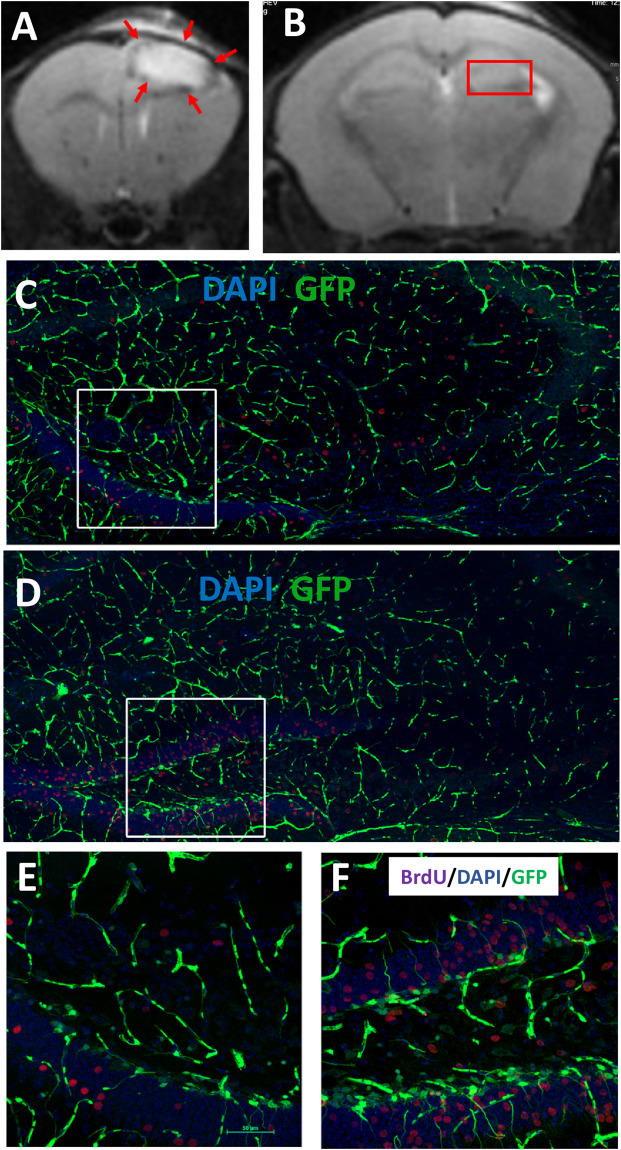
Evaluation of cell proliferation in GFP^+^ cells in the adult hippocampus 7 days after focal ischemia. T2-weighted MRI scans of coronal brains slices in the area of prefrontal cortex **(A)** and dentate gyrus **(B)** after 7 days’ stroke induction. The red arrows indicate the ischemic region. The red frame marks the area of the dentate gyrus. Proliferation of GFP^+^ cells in the hippocampus of control **(C)** or rS1/9-treated mice **(D)**. **(E,F)** Higher zoom of the area marked by a white box **(C,D)** presented in **(E,F)**. Nuclei were stained with DAPI (blue), immunostaining with antibody against BrdU (purple).

The object of the study was a genetically modified reporter line of mice in which stem and progenitor cells are characterized by the expression of a GFP located under the nestin promoter (nestin-GFP mice) (Mignone et al., 2016). Focal brain damage in the prefrontal cortex was modeled by a photo-induced occlusion of cortical vessels. As a result, a focal focus of ischemic damage was formed, including only the area of the prefrontal cortex (Figures 5A,B). The relationship between injured areas (in prefrontal cortex), rS1/9-injection site, and hippocampus is presented in serial MRI scans (Supplementary Figure 1). Intracerebral injection in the area of ischemic damage of rS1/9 microparticles in the volume of 2 μL or saline solution to control animals was performed 24 h after stroke simulation. Analysis of confocal images of brain slices on day 7 revealed the presence of GFP^+^ cells in the area of the DG (Figures 5D–F). Based on the morphology of these cells, it was possible to identify resting neural progenitors [quiescent neural progenitors (QNPs)], which have a triangular soma and an expanding apical process that crosses the granular cell layer and ends in a set of small sprouts in the molecular layer of the DG (Figure 5F). It is known that because of asymmetric mitosis division, QNPs divide to form amplifying neural progenitors (ANPs) which have a round or oval shape and very short sprouts but also are GFP^+^ (Supplementary Figure 2, red arrow). In the figure presented in Supplementary Figure 2, one can see that in some places it is very difficult to identify QNPs and ANPs based only on morphological criteria because of the close location of cells. The objective of this study was to qualitatively assess changes in the number of GFP^+^ cells in the area of the stem cell niche—the DG, in response to brain damage followed by the transplantation of rS1/9 microparticles to the area of damage. An increase in GFP^+^ cells in the subgranular zone of the hippocampus compared with the control group (Figures 5C,E) was observed in the group of animals that received rS1/9 injection in the area of ischemic injury (Figures 5D,F). Besides, we examined cell proliferation following a photothrombotic stroke when the mice received injections of BrdU (i.p., 100 μg/g body weight) 5 days after rS1/9 injection. The number of BrdU-positive cells in the DG of rS1/9-treated mice (Figures 5C–F) was significantly higher than in the control group mice (Figure 5E). The distribution of BrdU-positive cells in the subregions of the dentate gyrus was significantly different in both groups. BrdU-positive cells were located primarily in the granule cell layer and hilus of the dentate gyrus in rS1/9-treated mice (Figures 5E,F).

**FIGURE 6:**
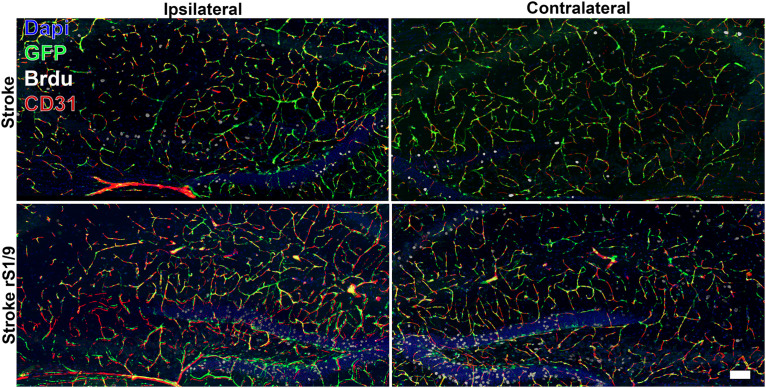
Evaluation of GFP^+^, CD31^+^, and BrdU^+^ cells in both contralateral and ipsilateral hippocampus 7 days after focal ischemia. Nuclei were stained with DAPI (blue), immunostaining with an antibody against BrdU (white) and CD31 (red), and nestin-GFP^+^ cells are green.

Additionally, we analyzed angiogenesis in the brain after the stroke and rS1/9 administration. The brain slices were stained with both BrdU (cell proliferation marker) and CD31 antibodies (vessel marker). We found that a significant part of nestin-positive cells (in all groups of animals) was colocalized in structures positively stained with antibodies to CD31. Thus, we can assume that some of the progenitor cells in the brain are derived from microvessel pericytes. Indeed, Nakata et al. (2017) also observed that nestin^+^ cells were adjacent to CD31^+^ cells in the ischemic zone suggesting the pericytic origin of neural progenitors. On the other hand, the majority of nestin-positive cells in the dentate gyrus area were not stained with CD31, so the stem cells located there did not belong to microvessels and pericytes. Analysis of nestin^+^ and BrdU^+^ cells revealed an increase in the proliferation of progenitor cells not only in the ipsilateral but also contralateral hemisphere (Figure 6). Quantitative analysis of BrdU^+^, nestin^+^, and CD31^+^ cells revealed a significant increase in BrdU^+^/nestin^+^ cells both in ipsilateral and contralateral hippocampus (Supplementary Figures 3, 4) in brains treated with rS1/9. However, rS1/9 treatment did not cause any increase in BrdU^+^/nestin^+^ cells in the ischemic area in cortex, as well as in peri-ischemic zone (Supplementary Figures 5, 6). Moreover, we found slight increase in CD31^+^ cells in hippocampus (Supplementary Figure 4) and penumbra area (Supplementary Figure 6) in mice after ischemia with rS1/9 treatment.

### Effect of rS1/9 Treatment on the Proportion of Different Types of Neural Cells in the Hippocampus

**FIGURE 7:**
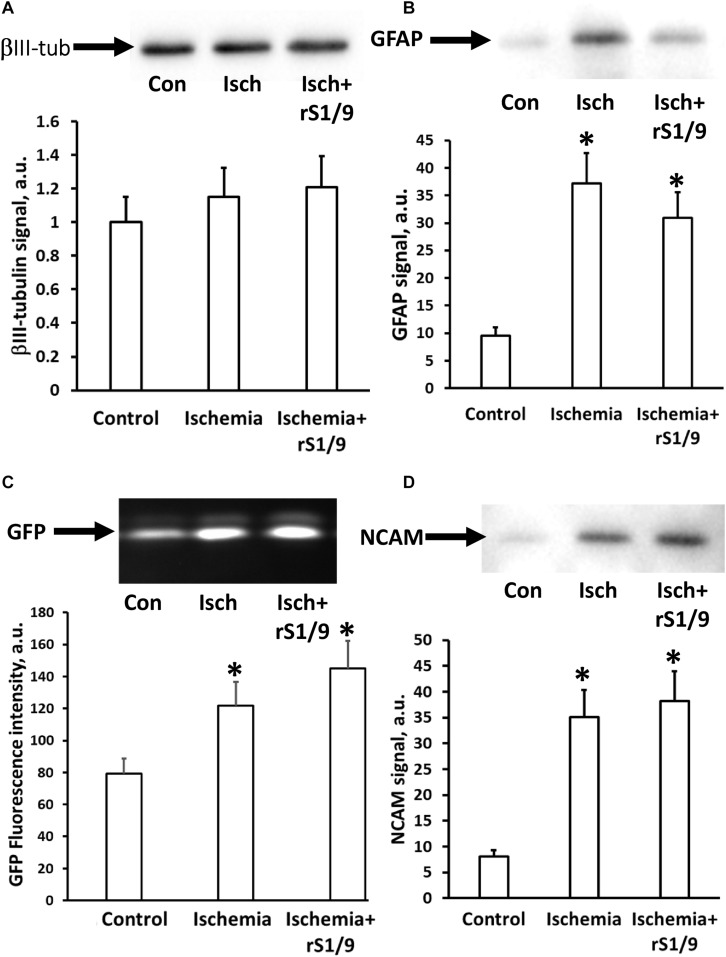
Changing the neuronal cell types in the hippocampus after rS1/9 treatment on the fourth day of stroke induction. **(A)** The level of βIII-tubulin, **(B)** glial fibrillary acidic protein (GFAP), **(C)** green fluorescent protein (GFP), and **(D)** neural cell adhesion molecule (NCAM) in hippocampus homogenates. Representative Western blots with averaged corresponding densitometry are presented. Number of animals was *n* = 4 per each experimental group. ^∗^*p* < 0.05, compared to control.

To determine alterations in the subtypes of cells in the hippocampus following brain ischemia and rS1/9 treatment, we analyzed the levels of a number of proteins that are markers of brain cells. Analysis of neuronal-specific tubulin showed no changes in the amount of βIII-tubulin in the hippocampus of mice on day 4 after brain ischemia. Injection of spidroin also did not affect the amount of tubulin, which indicates that there are no significant changes in the number of neurons in the DG of the hippocampus at these periods after ischemia (Figure 7A). At the same time, a significant increase in the number of GFAP (Figure 7B) was found which indicates the possible development of posttraumatic astrogliosis in the hippocampus region remote from the focus of ischemic damage. In the group of animals that received rS1/9, there was a tendency to decrease the number of GFAP signal, that is, a decrease in the pathological activation of astrocytes. The level of GFP also increased in groups of animals with ischemia or ischemia with spidroin treatment from 75.7 ± 1.6 in control to 122.1 ± 16.5 or 134.2 ± 15.8 a.u., correspondingly (Figure 7C, *p* < 0.05), which confirms our data on the increase in the number of neural progenitor cells obtained on brain slices in the hippocampus region. We analyzed the amount of NCAM in the hippocampus and found its significant increase after brain ischemia, as well as after injection of rS1/9 hydrogel in the area of ischemic damage (Figure 7D).

### Impact of Brain Ischemia and rS1/9 on Mitochondria of Neural Cells

**FIGURE 8:**
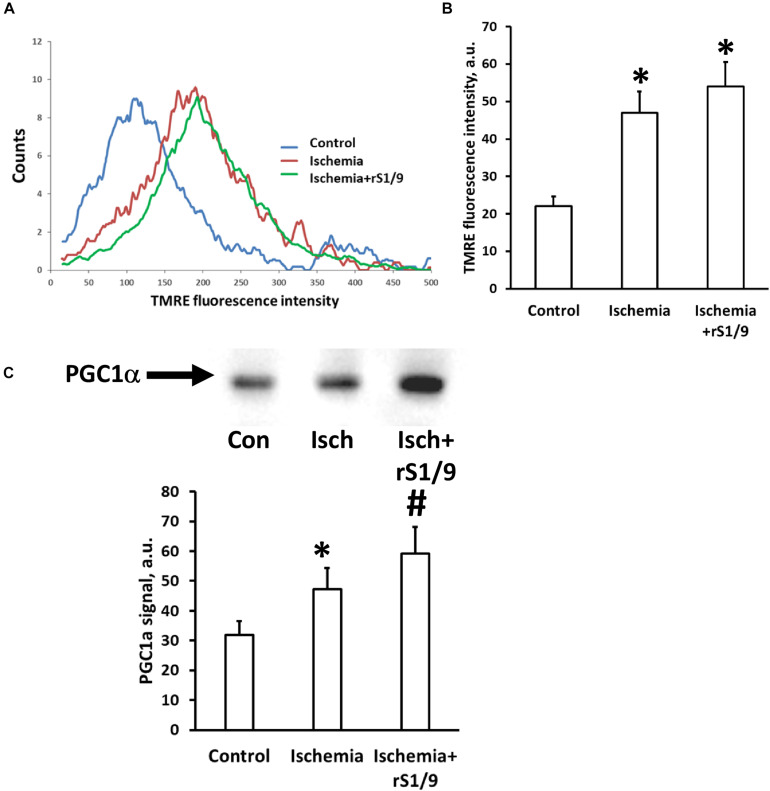
Effects of rS1/9 spidroin treatment on mitochondria of rat brain hippocampus after ischemic stroke. Mitochondrial membrane potential was estimated by TMRE loading in isolated neural cells immediately after trypsin digestion of mouse brain. Representative histograms of flow cytometry analysis of cell suspension **(A)** and quantification of mean TMRE fluorescence intensity **(B)**. Analysis of mitochondria biogenesis indicated by PGC-1α factor levels in hippocampal tissue **(C)**. ^∗^*p* < 0.05, compared to control, ^#^*p* < 0.05, compared to ischemia.

Mitochondria play a key role in the development of damage in the ischemic brain, and their normal functioning can be impaired by the action of damaging stimuli. Therefore, we analyzed the mitochondrial transmembrane potential in the hippocampus region of brain cells 7 days after ischemia. It was found that the intensity of the fluorescence of specific probe for the membrane potential, TMRE, significantly increases in cells isolated from the hippocampus after induction of photothrombosis in the prefrontal cortex compared to intact animals. However, when spidroin was administered to the area of ischemic injury, further enhancement of TMRE fluorescence was observed (Figures 8A,B). These changes may indicate either an increase in the transmembrane potential of mitochondria 7 days after ischemia or an increase in the number of mitochondria themselves. To distinguish these effects, we analyzed the levels of PGC1α, which is responsible for mitochondrial biogenesis and proliferation. It was found that the amount of this factor increases in hippocampal cells after brain ischemia from 31.9 ± 7.3 to 47.3 ± 8.6 a.u. (Figure 8C); thus, it gives evidence of a compensatory increase in mitochondrial biogenesis. The injection of spidroin enhanced this process (Figure 8C) and increased PGC1α levels up to 59.3 ± 9.8 a.u. Thus, an increase in the intensity of TMRE fluorescence in neural cells should be interpreted as an increase in the number of mitochondria, rather than as an increase in mitochondrial potential.

## Discussion

In this study, we analyzed the effect of microparticles from recombinant spidroin rS1/9 after its injection into the area of ischemic damage to the prefrontal cortex on the niche of stem cells in the DG of the hippocampus. It is well known that “new” (adult-born) neurons can be formed in the adult brain as a result of neurogenesis. In particular, such neurons were found in the DG of the hippocampus in rodents (Kempermann et al., 1997; Seki and Arai, 1999) and in humans (Knoth et al., 2010; Bergmann et al., 2015). These studies allow us to put forth a hypothesis about the possible participation of neurogenesis in the processes of brain plasticity and, as a result, in cognitive processes, as well as in the regeneration of brain tissue after damage. The peculiarity of “adult” neurogenesis is that the brain has already formed neurotransmitter systems that ensure the functioning of organized neural networks. The DG of the adult brain receives various direct and modulating neurotransmitter signals from several other structures including the prefrontal cortex (Jin and Maren, 2015; Nilssen et al., 2019). Damage to the prefrontal cortex is known to be associated with cognitive impairment due to impaired morphofunctional connections with the hippocampus (Hoover and Vertes, 2007; Silachev et al., 2009; Churchwell and Kesner, 2011; Bast et al., 2017). Activation of neurogenesis should promote functional recovery in this area of damage. We assumed that the delivery of a hydrogel based on the biologically active substance spidroin to the area of ischemic damage would promote the proliferation and survival of progenitor cells in the niche of brain stem cells (the DG).

The efficiency of using structural silk proteins from *B. mori* and *N. clavipes* for creating a matrix for neural tissue engineering has been demonstrated in numerous studies (Magaz et al., 2018; Salehi et al., 2020). Biomaterials based on silkworm fibroin are able to maintain the adhesion of neurons without adversely affecting their proliferation, morphology, and migration (Qu et al., 2010; Wei et al., 2013). In addition, fibroin scaffolds not only can support the adhesion and viability of cortical neurons but also carry a neuroprotective role demonstrated in the oxygen–glucose deprivation model (Moisenovich et al., 2019). It is known that the inclusion of RGD, GFPGER, and other peptide sequences in the structure of the material that mimic cell-binding motives of fibronectin, collagen, laminin, and other extracellular matrix proteins can regulate cell adhesion and, as a result, neuronal growth (Qu et al., 2010). The interaction of cellular receptors with such sequences mediates the influence of the microenvironment on cellular behavior. Neuronal membranes contain not only classical cell adhesion molecules, such as integrins and cadherins, but also neuron-specific cell adhesion molecules, such as NCAM (Kiryushko et al., 2004). NCAM is highly expressed in the central and peripheral nervous systems, where it plays an important functional role in cell adhesion, cell migration, and axon proliferation. In addition, NCAM is involved in the regulation of synaptic plasticity and recovery after brain damage (Park et al., 2018). Located on the surface of neurons, NCAM induces intracellular signaling pathways and cytoskeletal rearrangements in response to exposure to its extracellular domain (Wobst et al., 2015). Thus, NCAM-mediated cell adhesion is a critical step for triggering signaling events that lead to the growth of neurites (Maness and Schachner, 2007).

The recombinant analog of spidroin 1 rS1/9 used in our experiments contains 18 repeats of the NCAM-binding sequence GRGGL and is characterized by a high positive charge under physiological conditions, which is a critical property for NCAM-mediated adhesion (An et al., 2015). Films and microparticles obtained from rS1/9 were characterized by a complex surface and the presence of spontaneously formed micropores and submicropores (Figures 1A–D). It is known that the presence of pores can improve the diffusion of nutrients and oxygen in an artificial extracellular matrix. Also, during the formation of a neural network, the growth cone of the neurite is sensitive to surface elements, so that nanostructures and microstructure on the surface guide and modulate the growth of neurons. Moreover, it has been shown that neurites can penetrate pores with a diameter of 0.8–5 μm and extend the neurite coverage on the non-seeded side of the substrate, thereby increasing the volume of the neuronal network (George et al., 2018).

In our study, we demonstrated that rS1/9-based films provide better support for the growth of primary cortical neurons, including adhesion, neurite proliferation, and neural network formation, compared to fibroin-based films (Figures 2A–D). There was an increase in the intensity of NCAM-specific staining compared to fibroin (Figure 2E). This effect can be explained both by the content of a large number of NCAM-binding GRGGL sequences in the rS1/9 structure and a high positive charge and by the complex structure of the surface. These data are consistent with earlier studies that report a positive role of high charge and the presence of NCAM-binding motifs on the development of the neural network in cortical neuron culture (An et al., 2015). Thus, rS1/9-based materials can provide an attractive substrate for neurite germination and migration, which is one of the mechanisms of brain recovery after trauma (Park and Biederer, 2013).

It is known that solutions of silk proteins also carry biological activity and can interact not only with surface receptors but also penetrate the cell affecting the activation of signaling pathways, for example, the nuclear factor κB signaling pathway (Park et al., 2018). Based on the above facts, as well as taking into account the ability of rS1/9 to biodegrade in proteolytic (Krishnaji et al., 2013) and neutral (Moisenovich et al., 2011) conditions, we suggested that a suspension of microparticles added to damaged brain tissue may act as an intercellular matrix, and its products of hydrolysis may affect the growth of hippocampal nerve cells. To test this hypothesis, primary hippocampal neurons were cultured in a conditioned environment or in conditions where particles were isolated from direct contact with neurons by a membrane whose pore size prevented rS1/9 particles from directly entering the well with cells. In these experiments, we showed that the cultivation of hippocampal neurons in the presence of rS1/9 provides the formation of a larger number of longer neurites in terms of a cell, axon lengthening, and increasing its branching nodes (Figures 3D–G). The conditioned rS1/9 environment also had a positive effect on the length of neurites and axons but did not contribute to the formation of more neurites and axon branching points (Figures 3D–G). These results can be explained by the presence of products of hydrolysis of spidroin in the medium which carries multiple repeats of a neuron-specific GRGGL sequence recognized by neural progenitors (An et al., 2015). In case of culture in the Transwell culture system, neurons could receive rS1/9 hydrolysis products continuously formed from particles, in contrast to the conditioned environment, where the effect of dissolved spidroin or its hydrolysis products was bolus, which probably determined the difference in the results obtained. Thus, the injectable form of rS1/9 can affect neuronal growth without direct contact with cells, which is probably due to the formation of their biodegradation products, and it is important, given the distance of the injury zone from the hippocampus *in vivo*.

The next step of the work was to evaluate *in vivo* the effect of rS1/9 on the niche of progenitor cells in the DG. For this purpose, a reporter line of animals was used in which stem and progenitor cells were labeled with GFP associated with nestin expression which is a characteristic marker for this cell type (Bernal and Arranz, 2018). Nestin is expressed in the proliferative areas of the brain of embryos and adult mammals, and it is not found in differentiated cells of nervous tissue. Modeling of photothrombosis in the prefrontal cortex on day 7 led to an increase in the number of nestin-positive cells in the DG. Our data are consistent with a study in which with use of a similar line of mice it was demonstrated that traumatic brain injury in the sensorimotor cortex caused changes in proliferation of QNPs and ANPs in the acute phase following TBI (Gao et al., 2009). The hippocampus and prefrontal cortex are known to maintain functional interaction and anatomical connections and play a central role in various behavioral and cognitive functions. The ventral CA1 region of the hippocampus projects to the prelimbic medial prefrontal cortex and orbitomedial frontal cortex and the dorsal CA1 projects to the infralimbic and prelimbic parts of the prefrontal cortex (Sigurdsson and Duvarci, 2015). Because the prefrontal cortex has direct anatomical connections with the hippocampus (Jin and Maren, 2015), signal molecules such as rS1/9 and products of its degradation could directly enter the hippocampus and activate the processes of progenitor cell proliferation. Injection of rS1/9 microparticles into core ischemic damage resulted in a statistically significant increase in progenitor cell proliferation in the subgranular region of the DG. Additional labeling of proliferating BrdU cells on day 6 after rS1/9 injection revealed an increase in the content of BrdU^+^ cells in the granular layer of the DG. We also detected single double-positive GFP^+^ and BrdU^+^ cells. It was previously shown that only a small portion of GFP^+^ can be marked with BrdU after 24 h of pulse labeling (Filippov et al., 2003). We suggest that the injection of rS1/9 into the injury area can directly affect the proliferation of progenitor cells in the DG due to the diffusion of rS1/9 hydrolysis products from the injury area, which is consistent with our *in vitro* experiments.

As to the particular mechanisms of action of spidroin, it is necessary to keep in mind its effects on cell proliferation in contralateral DG where we found some increase in BrdU^+^ cells, although it was not as pronounced as in the ipsilateral hemisphere. It is also important to note that the contralateral hemisphere of animals treated with spidroin contains more CD31^+^ cells in the hippocampus region while only a trend for the increase was observed in the ipsilateral hemisphere. We supposed that in the latter compartment, the effect of increasing in CD31^+^ cells is similar to that in the contralateral hippocampus, but because of the more considerable variability of data apparently associated with the impact of ischemia, we were unable to achieve statistically significant values, contrary to the contralateral hemisphere which does not show high heterogeneity between samples. Also, the ipsilateral hemisphere contains much more nestin-positive and BrdU-positive cells. On the contrary, the contralateral hippocampus (since it was not exposed to damaging stimuli) mainly ignites neoangiogenesis caused by spidroin. These observations allow us to speculate that rS1/9 and its degradation products have not only a local, but also a systemic effect. Probably, these molecules are transported by the bloodstream to the contralateral hemisphere where they exert their influence on neurogenesis and angiogenesis. As has recently been shown, neural stem cells with their processes are directly in contact with the cells belonging to blood vessels and can capture substances from the blood (Licht et al., 2020).

We do not rule out that the injected spidroin gel could serve as a structural matrix through which progenitor cells could migrate to the area of damage. Reiner and Sapir (2018) showed that implanting a sponge coated with an adhesive factor in the injured neonatal brain supports the migration of neuroblasts and improves functional recovery. Additional analysis of markers of functional brain cells in the hippocampus by Western blotting showed that the marker of differentiated neurons βIII-tubulin remained unchanged. On the other hand, as a result of ischemic damage to the prefrontal cortex, the levels of GFAP increased, which may indicate the development of astrogliosis. Previously, remote cortical damage in the sensorimotor or prefrontal region has been shown to cause the proliferation of reactive astrocytes (Sandhir et al., 2008; Chin et al., 2013; Lee et al., 2020). Our results are also consistent with data from Gao and coauthors, who showed an increase in GFAP^+^ cells in the granule cell layer and hilus zone (Gao et al., 2009) in a similar mouse line during TBI modeling.

An interesting finding was the effect of brain ischemia in the prefrontal cortex on the status of mitochondria in hippocampal cells, as well as the effect of hydrogel therapy with spidroin on them. As we have shown, the distant effect of ischemic damage is realized via the activation of proliferative processes in the DG of the hippocampus and possibly by reactive astrogliosis. In line with these changes, we can interpret the change in the accumulation of potential-dependent mitochondrial dye in the cells of the DG, which increases after ischemia and after spidroin. An increase in TMRE fluorescence can be considered either as an increase in the mitochondrial transmembrane potential or as an increase in the number of mitochondria themselves. A partial answer to the question of which process prevails is given by analyzing the levels of PGC1α, which is an activator of the signaling pathway leading to mitochondrial biogenesis (Scarpulla, 2011). We found that the levels of PGC1α in the hippocampus 4 days after photothrombosis were increased, which indicates the activation of mitochondrial proliferation. We can consider these changes as a compensatory increase in the number of mitochondria in response to damage of brain tissue necessary for the adequate energy supply of stem and progenitor cell proliferation and differentiation (Cheng et al., 2012; de Oliveira Bristot et al., 2019).

We conclude that the stimulation of progenitor cells by rS1/9 and its effects on the growth of primary cortical neurons, their adhesion, neurite growth, and the formation of a neuronal network make this substance an excellent material for developing therapeutic strategies aimed at enhancing brain plasticity by influencing stem cell niches. Scaffolds formed from rS1/9 can also be used in regenerative medicine to enhance primary neuroprotection, resulting in reduced cell death directly in the injury area.

## Data Availability Statement

All datasets generated for this study are included in the article/Supplementary Material.

## Ethics Statement

The animal study was reviewed and approved by A. N. Belozersky Institute animal ethics committee (Protocol 5/18 from May 14, 2018).

## Author Contributions

MM, EP, DS, and DZ developed the concept, performed data analysis and manuscript writing, supervised the experiments, and analyzed the literature. DS, AM, AA, KS, VB, VD, AL, LZ, IP, and VB performed the experiments. All authors read and approved the final manuscript.

## Conflict of Interest

The authors declare that the research was conducted in the absence of any commercial or financial relationships that could be construed as a potential conflict of interest.
