# Supplementary material for: Effects of Recombinant Spidroin rS1/9 on Brain Neural Progenitors After Photothrombosis-Induced Ischemia
Source: Front Cell Dev Biol. 2020 Sep 8;8:823. doi: 10.3389/fcell.2020.00823 (PMC7505932; doi:10.3389/fcell.2020.00823)
Supplement: Supplementary file 1 [file Image_1.pdf]

Supplementary Material

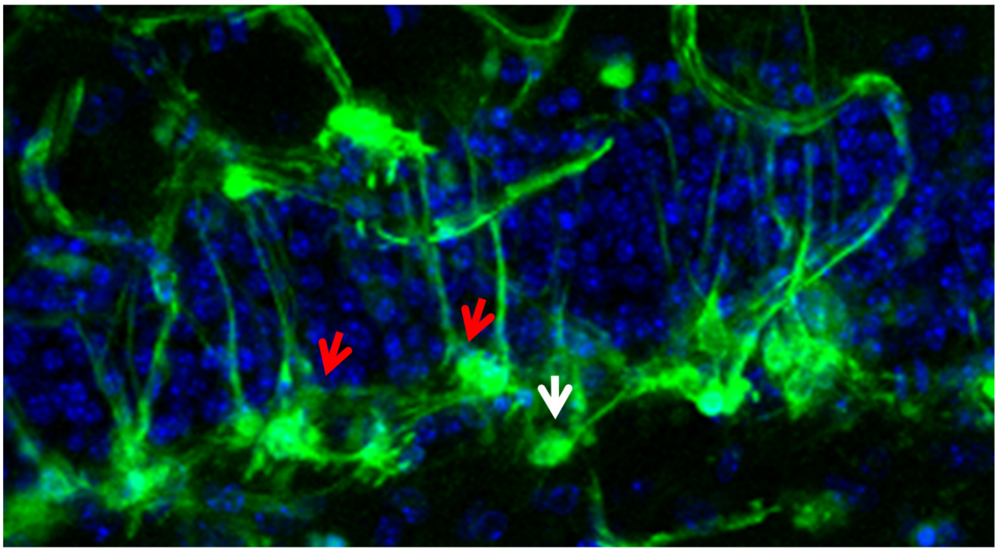

SUPPLEMENTARY FIGURE 1. Quiescent neural progenitors (QNPs) and amplifying neural progenitors (ANPs) were distinguishable in the hippocampus of transgenic mice Nestin-GFP. QNPs - marked with red arrows and ANPs - marked with white arrow.

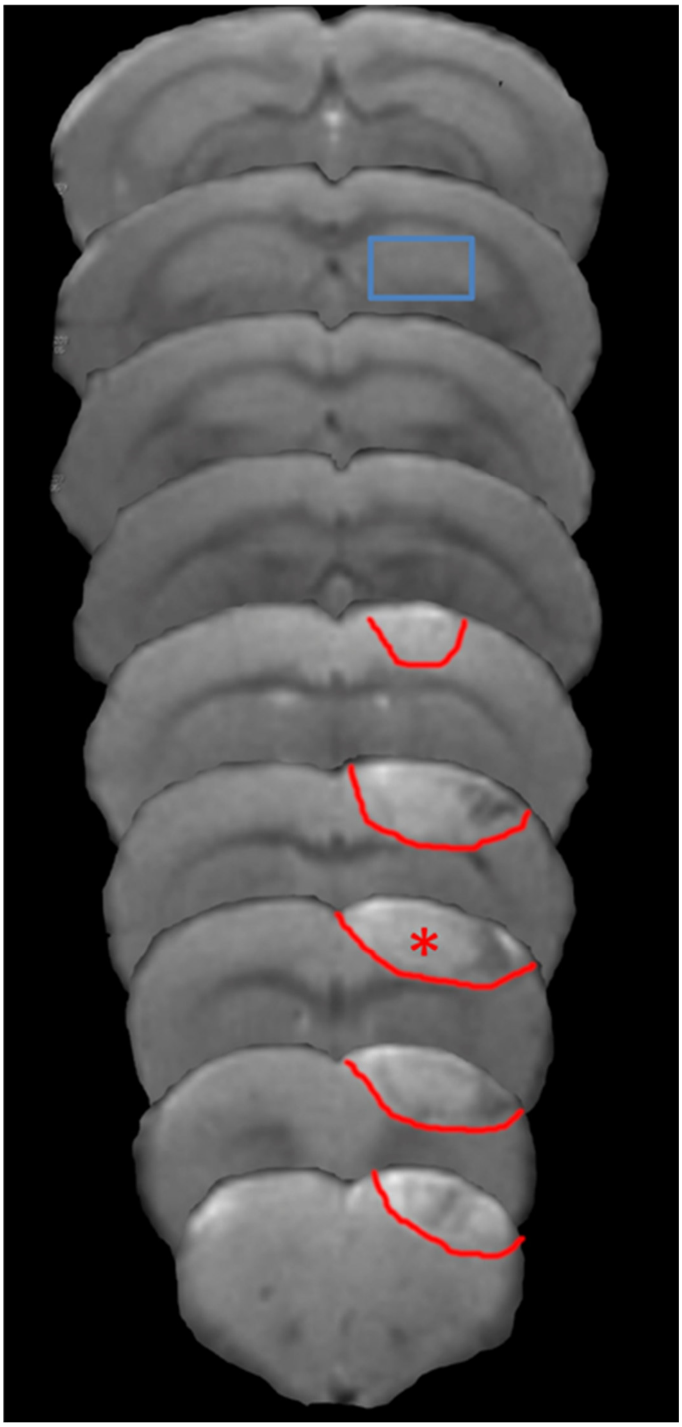

SUPPLEMENTARY FIGURE 2. Representative T2-weighted MR-images from coronal brain sections (0.5 mm thick), from caudal (top) towards rostral (bottom) obtained 7 days after photothrombosis. Hyperintensive regions refer to ischemic areas in prefrontal cortex (outlined red line). The blue frame indicated the area of the dentate gyrus. The red asterisk denotes spidroin injection site.

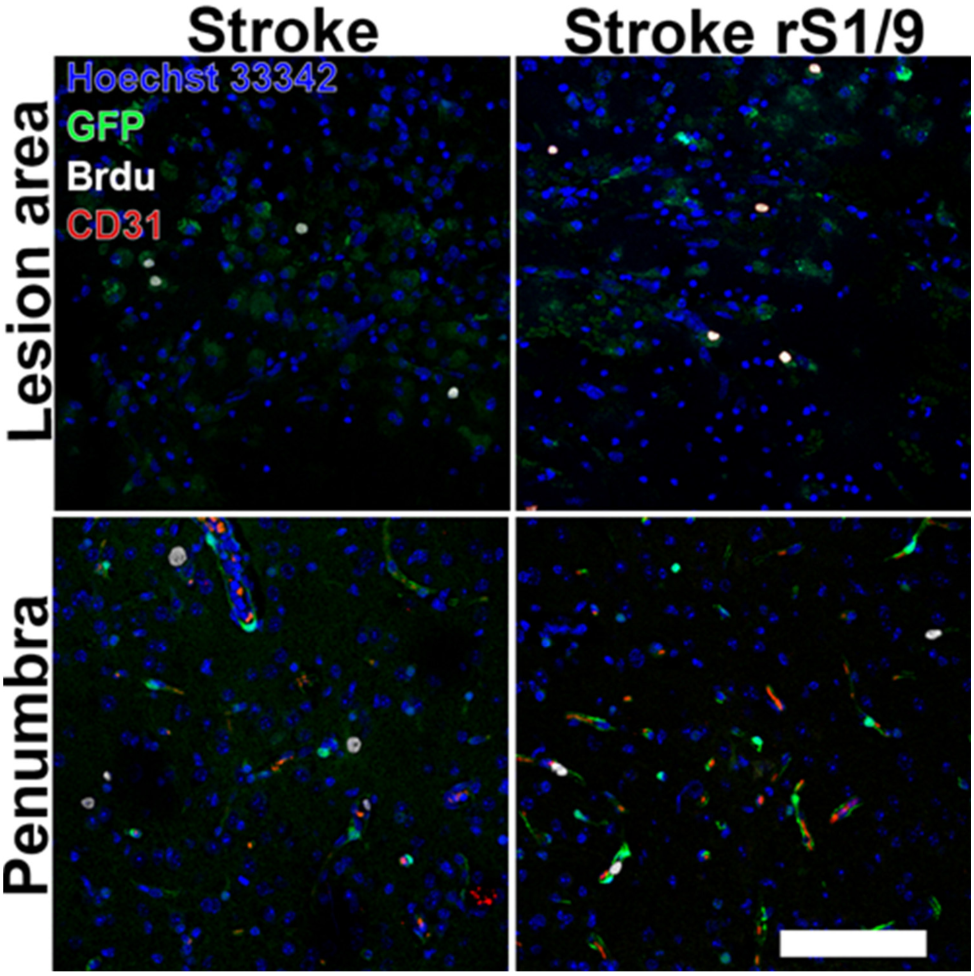

SUPPLEMENTARY FIGURE 3. Evaluation of GFP+, CD31+ and BrdU+ cells in the ischemic area and peri-infarct brain tissue 4 days after focal ischemia. Immunofluorescence staining was performed using paraffin-embedded tissue section. Nuclei were stained with Hoechst 33342 (blue), immunostaining with antibodies against BrdU (white) and CD31 (red), and nestin-GFP+ cells are green. Scale bar 50 µm.

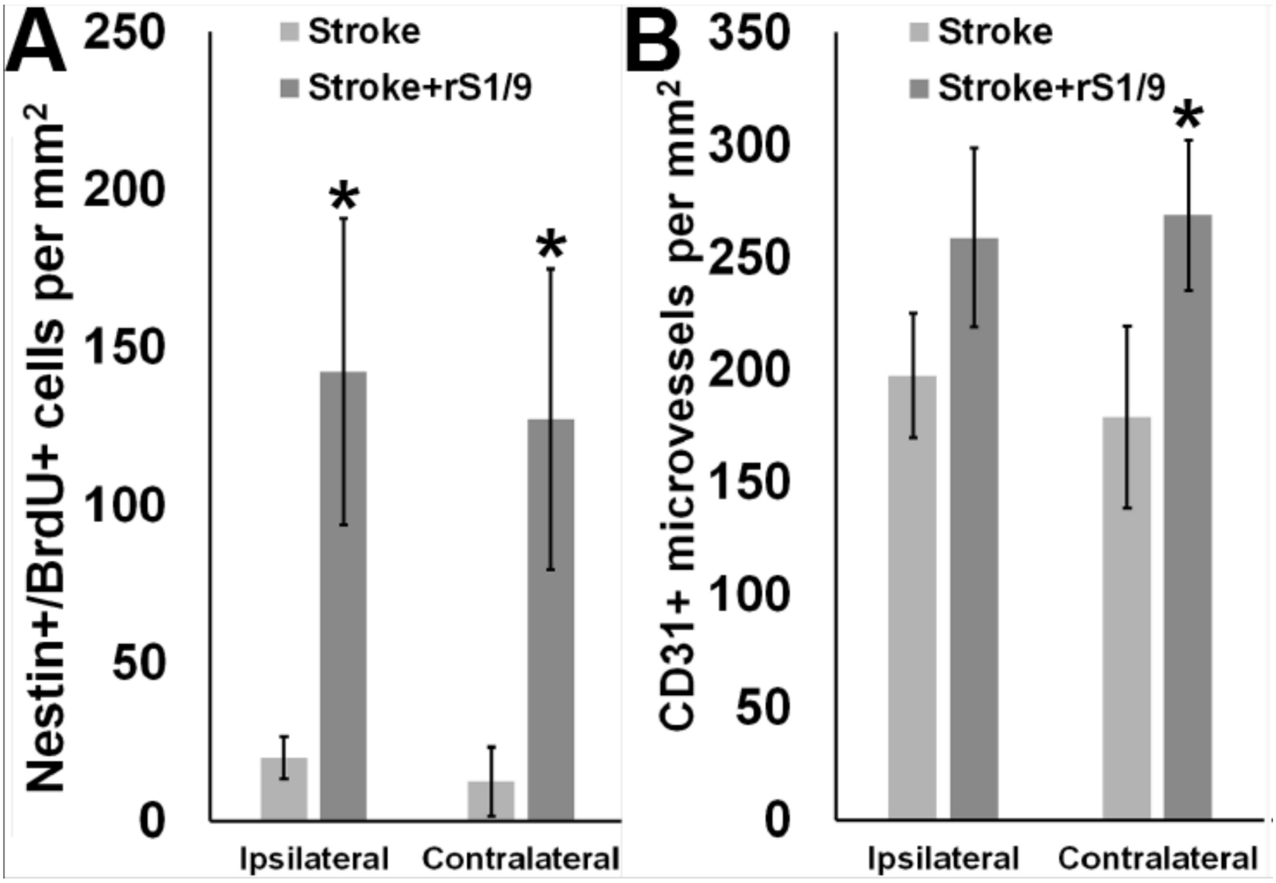

SUPPLEMENTARY FIGURE 4. Analysis of Nestin+/BrdU+ (A) and CD31+ (B) cells in the hippocampus of mice after brain ischemia or those treated with rS1/9. Data are presented for both the stroke-affected hemisphere and the contralateral hemisphere. Values are Mean ± SD of three independent experiments. * - significant difference vs. Stroke group ($p<0.05$).

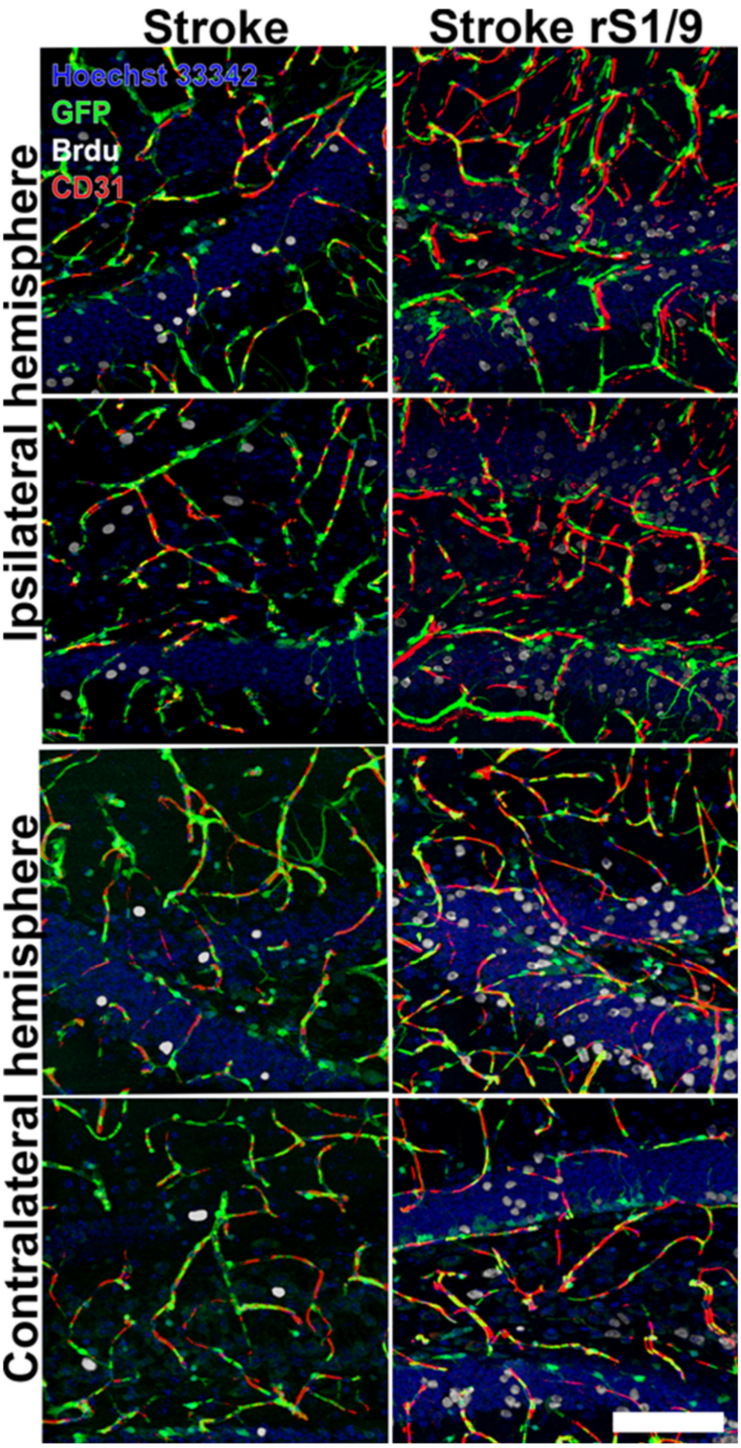

SUPPLEMENTARY FIGURE 5. Evaluation of GFP+, CD31+ and BrdU+ cells in ipsi- and contralateral hippocampus 7 days after focal ischemia. Immunofluorescence study was carried out on vibratome-sliced brain sections. Nuclei were stained with Hoechst 33342 (blue); immunostaining was performed using antibodies against BrdU (white), and CD31 (red) while nestin-GFP+ cells are shown in green. Scale bar 50 µm.

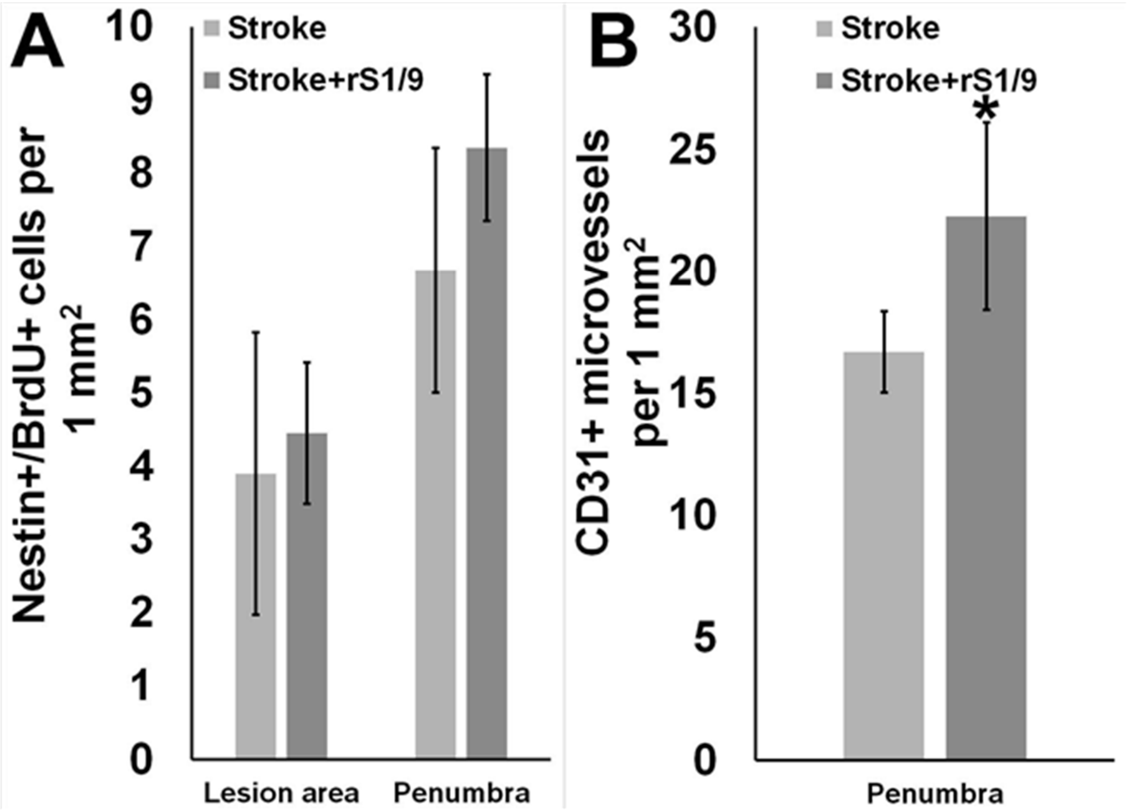

SUPPLEMENTARY FIGURE 6. Analysis of Nestin+/BrdU+ (A) and CD31+ (B) cells in the prefrontal cortex of the mouse after brain ischemia or those treated with rS1/9. Data are presented for both the lesion area and peri-infact zone (penumbra). Since CD31+ cells were not detected in the injury area, this data is presented only for the penumbra zone. Values are Mean ± SD of three independent experiments. * - significant difference vs. Stroke group ($p<0.05$).
